# Rapid, precise quantification of large DNA excisions and inversions by ddPCR

**DOI:** 10.1038/s41598-020-71742-z

**Published:** 2020-09-10

**Authors:** Hannah L. Watry, Carissa M. Feliciano, Ketrin Gjoni, Gou Takahashi, Yuichiro Miyaoka, Bruce R. Conklin, Luke M. Judge

**Affiliations:** 1 Gladstone Institute of Data Sciences and Biotechnology, San Francisco, CA USA; 2 Innovative Genomics Institute, Berkeley, CA USA; 3 Department of Pediatrics, UCSF, San Francisco, CA USA; 4 Regenerative Medicine Project, Tokyo Metropolitan Institute of Medical Science, Tokyo, Japan; 5 Department of Ophthalmology, UCSF, San Francisco, CA USA; 6 Department of Medicine, UCSF, San Francisco, CA USA

**Keywords:** Molecular biology, Genetic engineering

## Abstract

The excision of genomic sequences using paired CRISPR-Cas nucleases is a powerful tool to study gene function, create disease models and holds promise for therapeutic gene editing. However, our understanding of the factors that favor efficient excision is limited by the lack of a rapid, accurate measurement of DNA excision outcomes that is free of amplification bias. Here, we introduce ddXR (droplet digital PCR eXcision Reporter), a method that enables the accurate and sensitive detection of excisions and inversions independent of length. The method can be completed in a few hours without the need for next-generation sequencing. The ddXR method uncovered unexpectedly high rates of large (> 20 kb) excisions and inversions, while also revealing a surprisingly low dependence on linear distance, up to 170 kb. We further modified the method to measure precise repair of excision junctions and allele-specific excision, with important implications for disease modeling and therapeutic gene editing.

## Introduction

Gene editing using CRISPR-Cas9 or other nuclease systems in eukaryotic cells occurs via precisely targeted double-stranded DNA cleavage events, followed by repair by endogenous cellular machinery^1^. The Cas9 nuclease finds its target via a guide RNA (gRNA) with complementarity to the desired locus^2^. Many gene editing approaches rely on imprecise non-homologous end-joining (NHEJ) repair to create small insertions and deletions (indels) that disrupt a gene at a single cut site. Other approaches rely on homology-directed repair (HDR) to introduce a new sequence at the double-stranded break. Larger editing events can be produced by simultaneously delivering two nucleases targeted to different sequences on the same locus, which can lead to large deletions via excision of the intervening genomic sequence^3–7^. Generating excisions with paired CRISPR gRNAs is an attractive means to engineer complete loss of gene function, map regulatory regions, study 3D genome organization and model deletion-induced diseases. Furthermore, various therapeutic applications utilize paired gRNA to remove precise regions of DNA to induce alternate exon splicing, inactivate dominant disease alleles, remove toxic repeat expansions and delete viral integrations^8–15^. However, dual gRNA editing produces multiple editing outcomes, including excisions and inversions between the two cut sites, and indels at one or both sites^3,5^. There is currently no reliable approach to predict the relative frequency of these outcomes, or to measure it accurately and efficiently.

Small indels produced at individual target sites are routinely assessed via sequencing PCR amplicons by either Sanger or next generation sequencing (NGS), including by our group^16–20^. However, the quantification of excisions by amplicon sequencing or other measurements of amplicon abundance is complicated by amplification bias due to the inherent size difference between the amplicons of the excised and non-excised alleles, limiting this approach to very short excisions^8,16,17^. Detection of inversions requires additional specific primers, adding further complexity and risk of differential amplification of multiple amplicons even if they are designed to be similar in size. The extensive optimization required to validate every combination of primers for each editing experiment makes this an impractical approach. Unfortunately, the primary alternative approach to quantifying large excision events has been to isolate large numbers of cell clones for genotyping and sequencing each clone^3,5^. This process is slow and labor intensive, with sensitivity limited by the number of clones that can be analyzed. It is also limited to proliferative cell lines that can undergo clonal isolation. Whole-genome sequencing (WGS) has been used to measure the frequency of a small excision, but is expensive, low throughput and read depth limits its ability to detect infrequent events^21^. Targeted single molecule DNA sequencing could provide a useful alternative, but remains expensive, may suffer from differential selection of excised sequences and be limited by maximum read length^22,23^. UDiTaS, a unidirectional sequencing method, is able to quantify excisions and inversions in population samples without amplification bias. However, UDiTaS requires additional investment, including novel library preparation, NGS and downstream computational analysis^24^. None of these methods allow for rapid, low cost, reliable and length-independent quantification of excisions at endogenous loci in a heterogeneous population.

Here, we introduce ddXR (droplet digital PCR eXcision Reporter) to enable the sensitive and precise quantification of excisions and inversions without apparent length limitations. Droplet digital PCR (ddPCR) has several advantages over standard PCR for the detection of gene editing events. It is highly sensitive and quantitative^25^ and has previously been used to measure NHEJ and HDR editing outcomes^26–28^. Furthermore, by encapsulating target DNA molecules in individual droplets before amplification, it minimizes the problem of amplification bias. Finally, data can be analyzed immediately without downstream library preparation or computational expertise. The ddXR protocol can be completed the same day as DNA extraction and produces a gain of signal (GOS) that makes it possible to measure even rare excision and inversion events in mixed edited populations. We demonstrate the accuracy and consistency of this assay to detect excisions and inversions ranging from 91 bp to 172 kb in length. We also describe further modifications of the method to measure precise repair events and to measure allele-specific excision in a model of dominant genetic disease. The speed, simplicity and versatility of ddXR make it an ideal standard for the quantification of excisions and inversions in genome editing experiments.

## Results

### ddXR accurately detects excisions and inversions over broad length and frequency ranges

**Figure 1:**
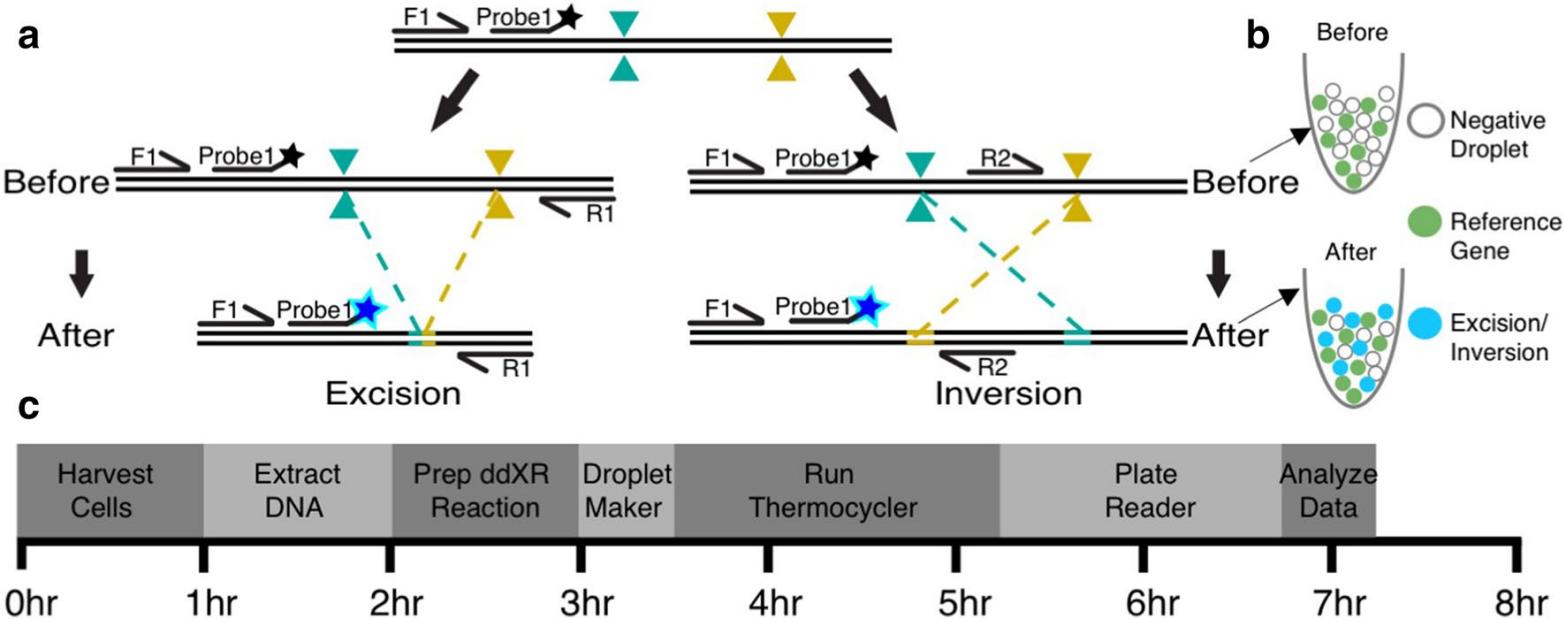
Overview of ddXR excision and inversion quantification methods. (**a**) Schematic of ddXR excision and inversion assay designs. Both inversion and excision occur in the same sample, but they are assayed in separate reactions. Note that the only variation between the excision and inversion assay is the reverse primer. Forward (F1) and reverse primers (R1, R2) are indicated by arrows along with FAM-conjugated fluorescent probe. Primers and probes to the reference gene are not shown. (**b**) Illustration of expected droplets detected before (upper) and after (lower) editing is performed. The number of excision or inversion positive droplets (blue) is normalized to the number of RPP30 positive droplets (green). RPP30 is a reference gene present at two copies in the human genome. (**c**) Workflow of DNA extraction and ddXR assay with time estimates.

We first tested our ddXR approach with pairs of Cas9/gRNA ribonucleoprotein (RNP) complexes targeting two genomic loci present at two copies in the reference human genome. We designed assays to produce a gain of signal measurable by ddPCR for excision and inversion occurring at each target locus (Fig. 1a,b). For excisions, we designed primers and FAM-labelled probes flanking the nuclease target sites so that they would be brought into close proximity after excision occurs, allowing for efficient amplification and activation of the fluorophore (Fig. 1a,b). We reasoned that unedited alleles would not produce a signal, as amplification of the intact sequence would be inefficient. For inversions, the same FAM probe and its associated primer were used, along with an alternate second primer located between the gRNA target sites and targeting the same strand as the first primer. Inversion reverses the orientation of the second primer and brings it into proximity with the first primer and probe, allowing for specific amplification (Fig. 1a). Combining this design with digital PCR technology allows amplification to occur at the single-molecule level, critical for minimizing amplification bias. Finally, we added an internal control assay for a reference gene, RPP30, also present at two copies in the human genome. The RPP30-specific probe was labelled with HEX, which allowed us to easily calculate the proportion of alleles with an excision or inversion in our target gene by normalizing the FAM signal to the HEX signal. The ddXR protocol can be completed in one day (Fig. 1c).

**Figure 2:**
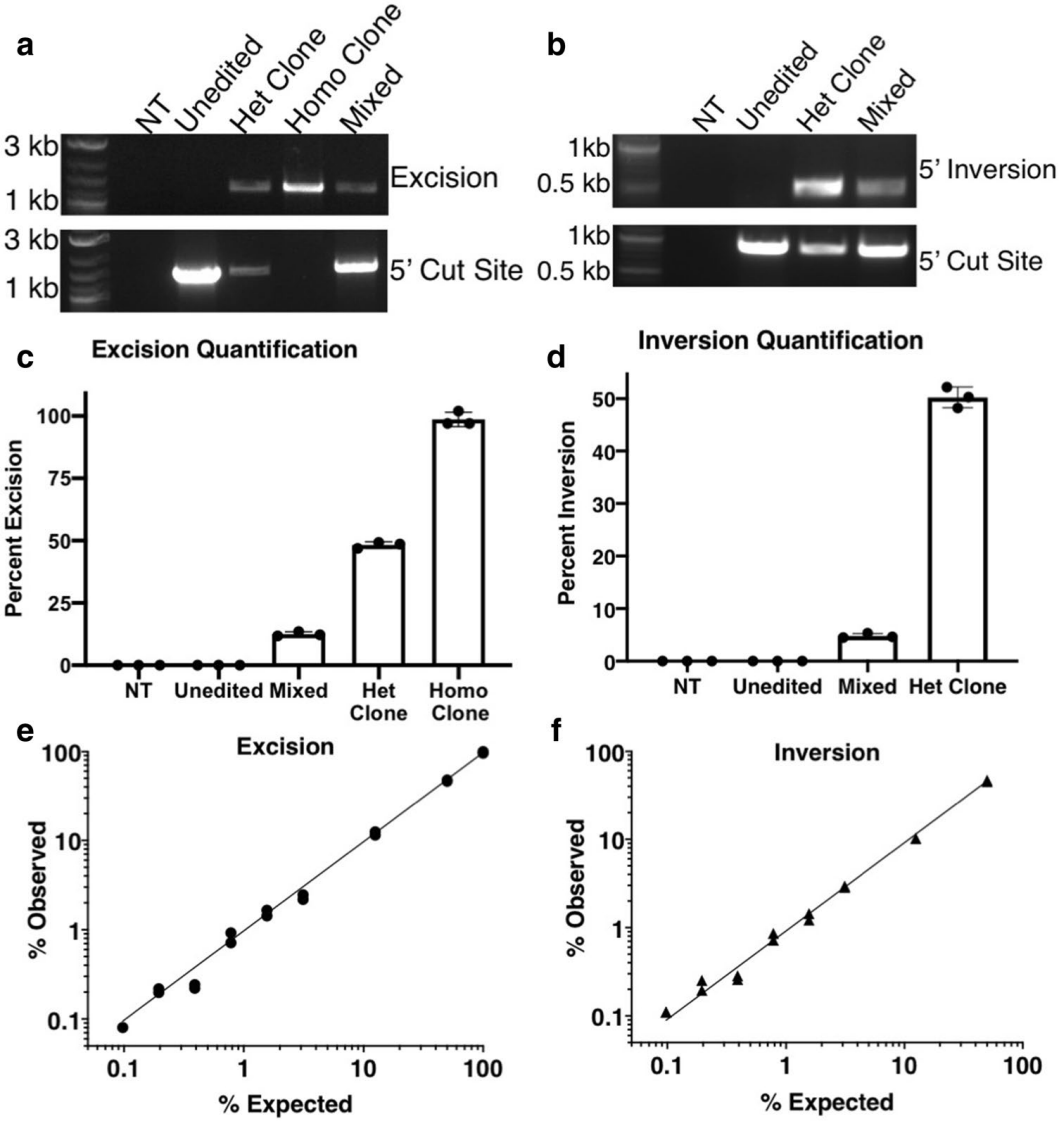
Validation of ddXR using clonal iPSC lines to measure excision and inversion. (**a**, **b**) Qualitative detection of excision/inversion and characterization of clonal cell lines used to validate assay. Edited clones were derived from previously characterized, karyotypically normal iPSC lines. PCR assays specific for a 4 kb excision on chromosome 8 (**a**) or 1.7 kb inversion on chromosome 7 (**b**) produced bands in mixed samples and clones. *NT* no template. Control PCR spanning 5′ cut site detects alleles without excision/inversion and produced bands in the unedited sample, heterozygous clones and mixed sample. We did not identify any clones with homozygous inversion. (**c**, **d**) Quantification of excision (**c**) and inversion (**d**) in mixed and clonal populations using ddXR assay. Values represent the percent of total alleles with excision/inversion and are mean of three replicates ± S.D. (**e**, **f**) ddXR quantification of standard samples with defined frequency of excision (**e**) and inversion (**f**). Samples prepared by serial dilution of clonal DNA with the unedited parental line. Data include two independent replicates of each dilution and are presented as the percentage of alleles with excision/inversion detected by ddXR. Note that in (**f**) the maximum inversion frequency is 50% as it was prepared using genomic DNA from a heterozygous clone. R^2^ = 0.999 (**e**) and 0.998 (**f**).

To validate the method, we transfected euploid, human iPSCs with the two pairs of RNP complexes. One pair was located 4.09 kb apart on chromosome 8 and the other 1.7 kb apart on chromosome 7. We isolated clones with heterozygous and homozygous deletion of 4.09 kb on chromosome 8 and heterozygous inversion of 1.7 kb on chromosome 7, as determined by traditional PCR (Fig. 2a,b). Based on the FAM-to-HEX ratio, we obtained modest levels of excision (12.5%) and inversion (4.8%) in the edited iPSC populations. Clonal iPSC lines produced expected ratios of excised and inverted alleles—48.3% and 98.7% in heterozygous and homozygous excision lines, respectively, and 50.2% in a heterozygous inversion line (Fig. 2c,d). We isolated additional clonal excision lines, with excisions on chromosomes 7 and 8, and again detected heterozygous excisions with the expected frequency (53.5% and 47.8%, respectively; Fig. S1). Next, we asked whether locating the probe at the 5′ or 3′ end of an excision or inversion would affect the results of the assay. We tested this in the clone with the largest excision (14 kb on chromosome 1) and found no significant difference in excision frequency measured by the two probes (Fig. S2a,b). We also compared 5′ and 3′ assays for two different inversions in polyclonal populations and obtained equivalent results in both cases (Fig. S2c,d). We did not observe false positive signals from unedited control samples in any of these assays, although we did detect a high false positive rate when generating very short excisions (< 200 bp). This was caused by efficient amplification of the non-excised alleles and could be easily removed by an internal restriction digest (Fig. S3).

To evaluate the dynamic range of ddXR, we generated genomic DNA samples with known proportions of either excised or inverted alleles. We prepared two independent serial dilutions of genomic DNA from two sets of edited clones (homozygous excision and heterozygous inversion clones) with genomic DNA from the unedited parental clone. We tested each dilution series with the ddXR assay and obtained results that were highly accurate, even when measuring samples with excision or inversion frequencies as low as 0.1% (Fig. 2e,f). A previously reported approach for quantifying excision frequency with ddPCR used the loss of signal (LOS) of an internal probe^29^. We repeated the analysis of the standardized excision samples using a LOS ddPCR assay. In this case, there was reduced accuracy and linearity, with root mean square error over four times higher than that for the corresponding ddXR assay (Fig. S4). The LOS method performed particularly poorly at lower concentrations of excision alleles.

We next compared the frequency of excisions and inversions measured by ddXR to those determined by PCR genotyping of individual iPSC clones. We handpicked 48 colonies and genotyped the surviving clones from four distinct paired RNP transfections (Table S1). We then compared the proportion of excision alleles based on clone genotyping to the ddXR quantification from the same transfection. There was reasonable agreement between the methods, with one experiment producing a modest discrepancy between ddXR and clone genotyping (6.8% vs. 11.5% respectively, Table S1). However, there was variability in the number of surviving clones between experiments, with low total numbers of positive clones limiting the statistical power of this approach. These results provided independent confirmation of the excision and inversion rates measured by ddXR, while highlighting the enhanced speed, precision and cost effectiveness of ddXR.

### Excision and inversion correlate poorly with length

**Figure 3:**
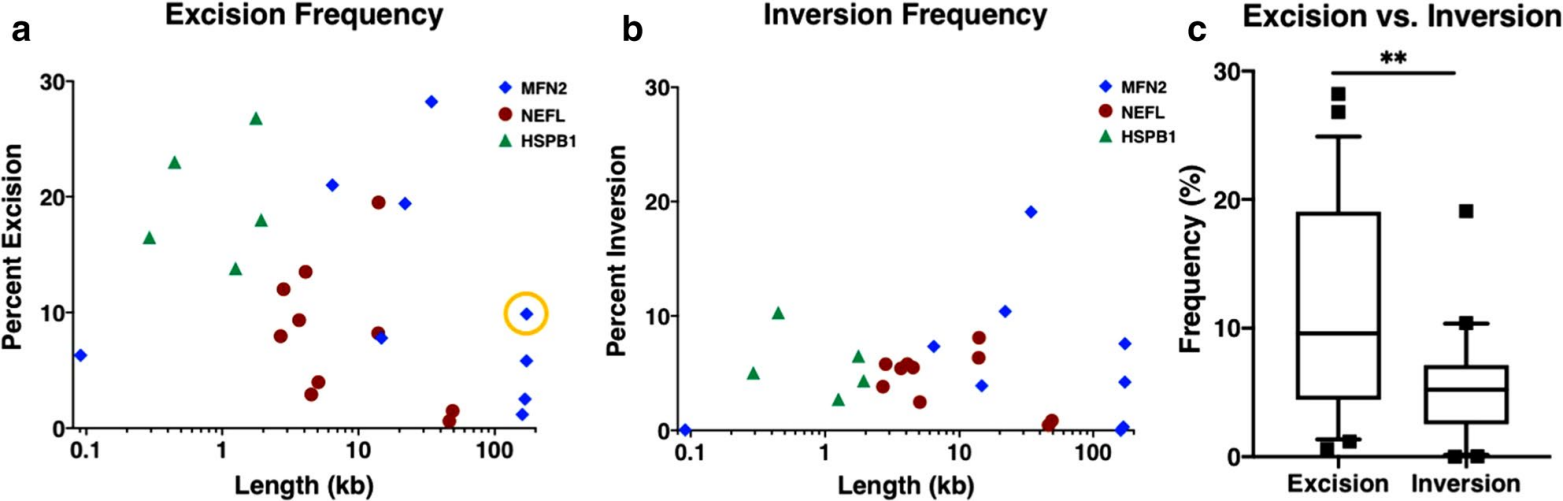
Quantification of excision and inversion frequencies versus linear distance between cut sites. (**a**, **b**) Dot plots of all excisions (**a**) and inversions (**b**) measured at three loci with varying linear distance between paired CRISPR-Cas9 target sites (23 unique combinations). Color and shape of points indicate locus. Pearson correlation suggests that excision is negatively correlated with increasing length, but with only borderline significance. r = −0.409, *p* = 0.047 (**a**) and detected no correlation for inversions, r = −0.216, *p* = 0.310 (**b**). Circled excision rate in (**a**) was validated by FISH (Fig. S6). (**c**) Box plot comparing overall frequency of excisions and inversions across the entire dataset as percentage of total alleles. Whiskers extend to 10th and 90th percentile. Outliers are marked with black squares. Median_excision_ = 9.589%, Median_inversion_ = 5.230%, *p* = 0.0015.

The factors that influence excision efficiency are not well understood, although it is widely believed that there is a strong negative correlation with increasing linear distance between cut sites^3^. We applied ddXR to investigate this possibility in three genes that differ in size, numbers of exons, chromosome and expression patterns. We designed gRNAs and ddXR assays and transfected our wild type control iPSC line with various pairs of RNP complexes targeting each locus, with a linear distance between target sites ranging from 91 bp to 172 kb (Tables S2, S3). Genomic DNA was isolated from each transfected population and assayed for both excision and inversion (Figs. 3a,b, S5a). Interestingly, we saw wide variation in excision and inversion frequencies across the full range of linear distance between cut sites, with only a weak correlation between excision and linear distance and no correlation between inversion and linear distance (Fig. 3a,b). We additionally observed a significantly lower overall rate of inversions compared to excisions (Figs. 3c, S5e). We confirmed that these results were not cell line specific by performing a similar analysis on combined data from three additional iPSC lines; one from a healthy control and two from patients with neurological disease (Fig. S5b–e). The longest excision (172 kb on chromosome 1) occurred at an unexpectedly high frequency (9.87%, Fig. 3a), presenting the opportunity to validate this result by fluorescent in-situ hybridization (FISH). FISH was performed using one probe targeting the deleted fragment and a second control probe targeting the same chromosome 5.5 Mb away. Chromosomes containing the excision were identified by positive signal for the control probe but negative signal for the target probe, which occurred in 10% of alleles in the transfected population (Fig. S6a,c). This was highly consistent with the excision frequency of 9.87% determined by ddXR on the same sample (Fig. S6a). No chromosomes showed loss of target signal in the unedited control by FISH (Fig. S6b).

### ddXR distinguishes between precise and imprecise excisions

**Figure 4:**
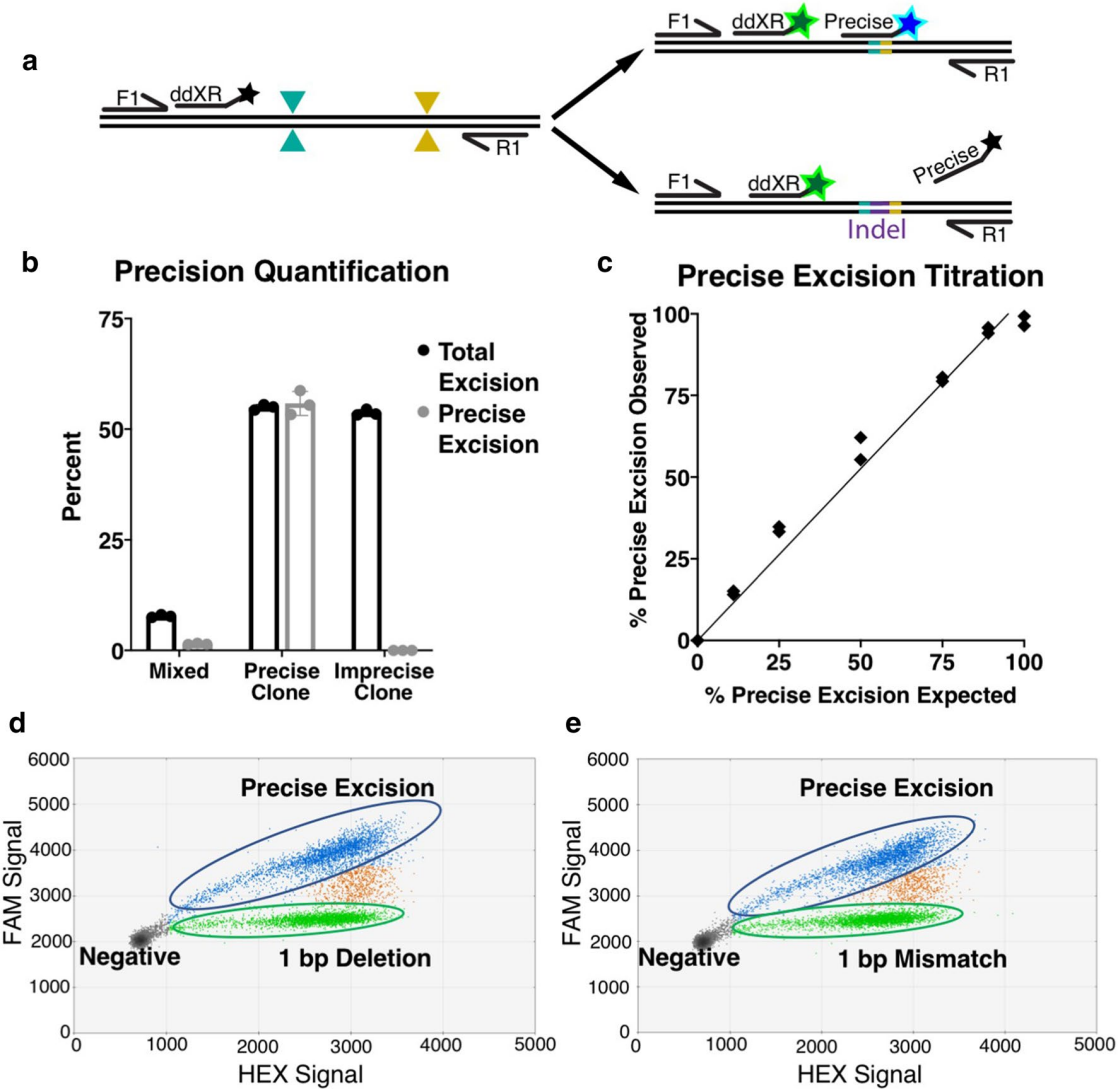
Design and validation of precise excision assay. (**a**) Schematic of precise excision assay. A probe is designed to align to the predicted excision junction. The probe can be duplexed with the general ddXR probe or a reference probe for normalization (not shown). (**b**) Percent of alleles with total and precise excision in population and clonal lines, presented as mean of triplicate assays ± S.D. (**c**) ddXR quantification in samples with defined frequency of precise excision. Duplicate samples were prepared by serial dilution of precise excision clonal DNA with imprecise excision clonal DNA. Presented as percent of excision alleles with precise repair versus expected, R^2^ = 0.98. (**d**, **e**) 2D plots of genomic DNA spiked with 1:1 mixtures of *HPRT1* excision reporter plasmids with precise and imprecise excision. Alleles with precise excision are positive for FAM and HEX (blue) while alleles with either type of imprecise excision are positive for HEX only (green). Measured ratio of precise to 1 bp mismatch was 0.952 (**d**, Poisson range 0.996–0.909) and of precise to 1 bp deletion was 0.919 (**e**, Poisson range 0.961–0.878).

When sequencing excision junctions, we noticed that many samples had small insertions or deletions at the repair junction, consistent with other types of NHEJ repair. The ability to produce precise excision events is an important tool for studies of biological function as well as gene therapy^6,15^. To determine the frequency of these events, we designed an assay to detect precise excision repair, where the two cut sites are ligated together without additional insertions or deletions. This assay uses the same primers as the general ddXR excision quantification assay, but a probe that spans the predicted junction (Fig. 4a). To test the assay, we isolated two clonal iPSC lines each with a 14.7 kb excision on chromosome 1, one matching the predicted excision repair (precise excision clone) and one with an insertion of 23 bp at the excision junction (imprecise excision clone) (Fig. S7a). The probe designed to detect the precise excision event gave equivalent results to the original ddXR assay in the precise excision clone and gave no measurable signal in the imprecise excision clone. In a mixed population of edited alleles, an average of 1.5% had a precise excision (Fig. 4b). This sample had an overall excision rate of 7.8% (Fig. 4b), suggesting that approximately 19% of all excisions underwent precise repair. We further validated this assay by performing a serial dilution of genomic DNA from the precise excision clone with genomic DNA from the imprecise excision clone and quantifying precise and total excision in each sample. The measured ratio of precise/total excision in each sample showed good agreement with the known ratio across the linear range (R^2^ > 0.98, Fig. 4c).

Next, we tested whether this approach could discriminate precise repair events with single nucleotide sensitivity. To do this in a controlled manner, we designed three reporter plasmids containing a portion of the *HPRT1* gene sequence mimicking different repair outcomes after excision of a 4.45 kb fragment: (1) precise excision repair, (2) deletion of a single nucleotide at the excision junction and (3) substitution of a single nucleotide at the excision junction. We spiked genomic DNA from our wild type iPSC line with each plasmid individually, as well as mixtures of equal concentrations of the precise excision plasmid paired with each imprecise version. We subjected each sample to a duplexed assay with a general ddXR excision probe and a precise excision probe labelled with different fluorophores (Fig. 4a). As predicted, precise excision was detected as a distinct population that was positive for both fluorophores, while both versions of imprecise excision were positive for the HEX fluorophore only (Figs. 4d,e, S7b–d). The measured ratios for the 1:1 mixtures of precise repair to single nucleotide substitution and precise repair to single nucleotide deletion showed good agreement at 0.919 and 0.952, respectively. Thus, ddXR serves as a flexible platform to quantify general and precise excision events.

### ddXR detects allele-specific excisions

**Figure 5:**
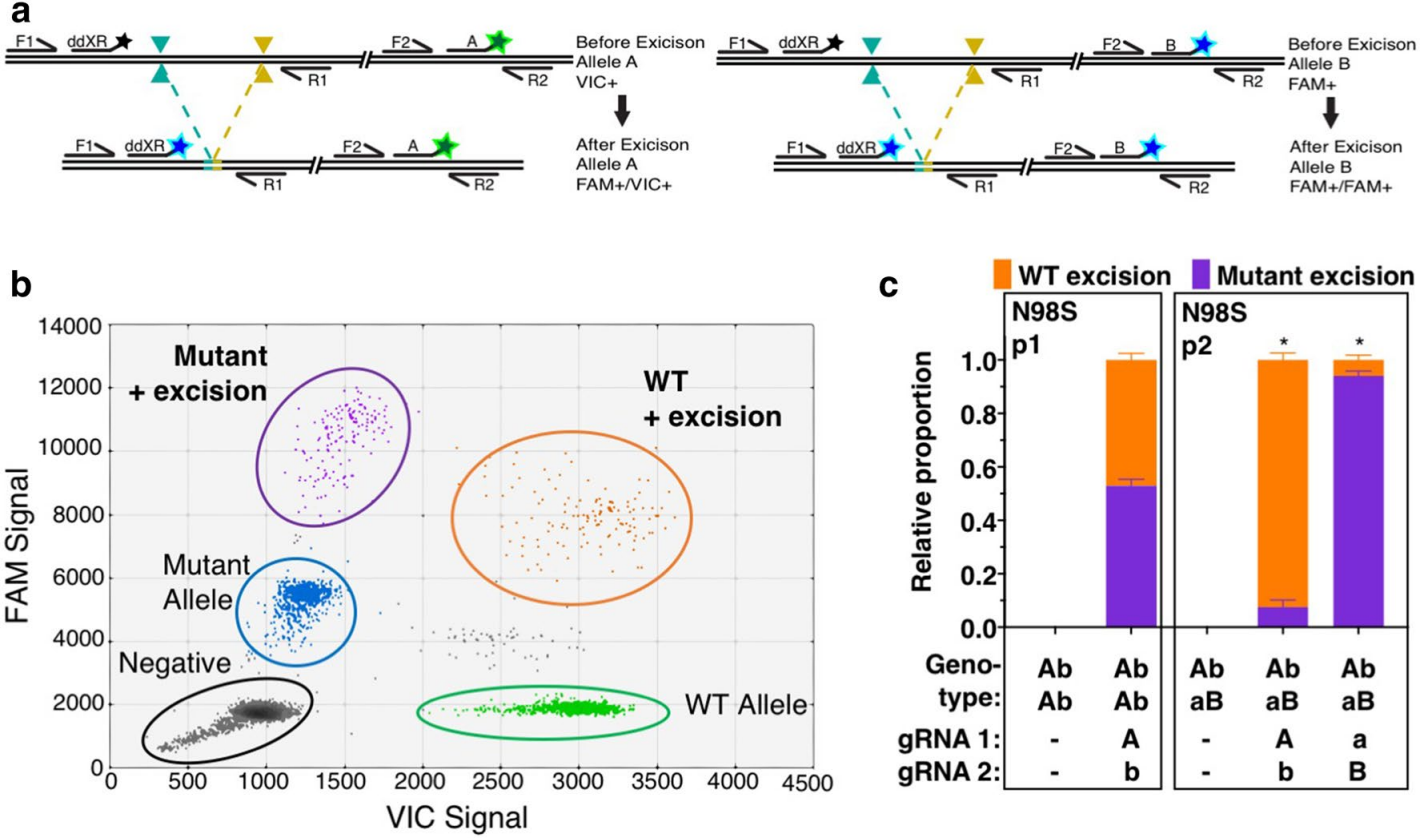
Design and validation of allele specificity assay. (**a**) Schematic demonstrating allele-specific assay design. ddXR primers (F1, R1) and probe are multiplexed with allele discrimination primers (F2, R2) and probes (A = VIC, green and B = FAM, blue) targeting a heterozygous SNP near the excision site. Excision on either allele produces a distinct double-positive signal (A = FAM–VIC, left and B = FAM–FAM, right). (**b**) Representative data from heterozygous *NEFL*-N98S iPSCs with excision on both alleles. Unedited alleles produce single-positive VIC (wild type, green) or FAM (mutant, blue) signal. Double-positive signals for excision on each allele are identified by orange (FAM–VIC, wild type allele) or purple (FAM–FAM, mutant allele). (**c**) Quantification of allele-specific excision in polyclonal edited populations in two patient iPSC lines, presented as proportion of FAM–VIC versus FAM–FAM signal. In a cell line homozygous for the gRNA sequence (N98S p1), excision occurred on both alleles, with 52.9% linked to the mutant. In a cell line heterozygous for SNPs in the protospacer sequence (N98S p2), a mean of 92.5% of excisions occurred on the WT allele when it was targeted and mean of 94% occurred on the mutant allele when it was targeted. Values are the mean of three biological replicates ± S.D. Unedited controls showed no signal in both patient lines. *Indicates significant difference between mutant and WT excision with *p* value < 0.00001.

Allele-specific excision to delete dominant disease alleles is a promising therapeutic gene editing strategy^12^. A dominant missense mutation in *NEFL* causes a severe neuropathy that can be modeled in iPSCs to test this approach^30^. We modified ddXR to correlate excisions with specific alleles by adapting a ddPCR method previously developed for phasing SNPs on the same chromosome, using the linkage of two probes on the same molecule to produce double-positive droplets in the ddPCR readout^31^. We designed a pair of allele-discrimination probes targeting the heterozygous N98S mutation in *NEFL* (mutant-specific probe = FAM, wild type = VIC) and multiplexed them with the ddXR excision assay (Fig. 5a). We tested this assay on N98S patient-derived iPSCs transfected with a pair of RNPs not designed to be specific for either allele. In this sample, we identified FAM–FAM and FAM–VIC double positive droplets representing excision linked to either the mutant or wild type allele, respectively (Fig. 5b). As expected, we observed similar rates of editing at each allele in the population of edited cells (Fig. S8f). We next isolated iPSC clones from the edited population and used the allele-specific ddXR assay to identify clones in which excision occurred on a specific allele. Clones with heterozygous excision linked to either mutant or wild type allele were identified by the presence of double-positive droplets, either FAM–FAM or FAM–VIC, respectively (Fig. S8f). A single homozygous clone with excision on both alleles was identified by presence of both double-positive signals (Fig. S8d).

Finally, we tested whether this assay could discriminate allele-specific excision occurring in a polyclonal population shortly after editing. To accomplish this, we generated an iPSC line from a second patient with the N98S mutation who had multiple heterozygous non-coding SNPs in the *NEFL* gene. We designed gRNAs to be specific for the major and minor alleles at two different sites of variation, which we refer to as *A/a* and *B/b* for simplicity. We were able to impute the phasing of the two SNPs with high confidence to identify pairs of gRNA that would target each haplotype. Patient 1 was homozygous for haplotype *Ab* and patient 2 was heterozygous *Ab/aB*. Phasing of the disease-causing mutation was unknown as it was presumed to be a de novo event. We transfected both N98S cell lines with pairs of RNP targeting the haplotype(s) present in each cell line and performed the allele-specific ddXR assay 48 h later. As expected, excision occurred at similar frequencies at the wild type and mutant allele for patient 1. However, excision was > 90% specific when targeting either allele for patient 2, revealing in the process that the disease mutation was linked to haplotype *aB* (Fig. 5c). These data confirm that heterozygous SNPs adjacent to the site of an excision can be incorporated into the ddXR design to determine whether an excision is specific for a particular allele in both clonal and polyclonal samples.

## Discussion

Here, we show that ddXR provides an accurate and length-independent method for quantifying the frequency of inversions and excisions in genome editing experiments. This is especially important for applications that require deletions larger than a few hundred base pairs. While excision length is limited in studies that rely on NGS for quantification^8,16,17^, we are able to quantify excisions up to 172 kb and have not yet reached an upper limit to the length of excision or inversion detectable by ddXR. We compared ddXR to other methods, including clone genotyping and FISH, which produced comparable but lower throughput results. We further showed that ddXR outperforms a LOS ddPCR assay, displaying higher precision across a wide range of editing efficiencies. Therefore, ddXR fills a need in the field for a flexible and rapid screening method to quantify excisions and inversions. We performed all of the assays and validation in well-characterized, euploid human iPSC lines, making our results maximally relevant to human gene editing.

Our modifications for the quantification of precise repair and allele specificity demonstrate the versatility of this assay in different genetic contexts (Table 1). The ability to design and carry out assays for such varied editing outcomes also points to the consistency of the assay. Though there are some design constraints for the sequences of the probes and primers, we have not yet encountered an excision for which we were unable to design an effective assay. However, other rearrangements such as duplications, translocations, and larger than expected deletions (the latter suggested by our FISH results and others’ reports^32–34^) cannot be excluded without other detection methods such as Southern blot, microarray, long-read sequencing, or FISH mapping. Although a single ddXR assay cannot measure all possible excision sizes and rearrangements, further adjustments, similar to the inversion assay, can be easily designed to quantify specific rearrangements of interest once they are defined. It is likely that other modifications will further expand the scope of ddXR. For example, a current limitation of the assay is that we cannot distinguish between heterozygous and homozygous excisions or inversions in a polyclonal sample, as all of the alleles are assayed in bulk. Single-cell modifications of the protocol would be a welcome future improvement.

**Table 1:** Summary of ddXR assays and modifications.

Assay	Primer Design	Probe design	Control	Notes
ddXR—Excision	Forward and reverse primers flank excision junction	Flanking either 5′ or 3′ target site	Genomic reference (e.g. RPP30)	Add restriction digest for excisions < 200 bp
ddXR—Inversion	Two forward primers (2nd replaces reverse excision primer, internal to cut sites)	Same as excision	Genomic reference (e.g. RPP30)	
Precision ddXR	Same as excision/inversion	Spans predicted repair junction	Genomic reference *or *general ddXR	Normalize to RPP30 for absolute *or* ddXR for relative
Allele-specific ddXR	Same as excision/inversion	Same as excision/inversion	Allele-discrimination to nearby SNP	Double-positive droplets identify linkage to SNP

An important practical application of our method will be the isolation of clonal cell lines with specific desired editing outcomes. For example, the ability to easily determine the frequency of the desired event informs the number of clones that need to be isolated and genotyped in order to identify a positive clone. Furthermore, different conditions could be rapidly screened to identify those that produce the highest frequency of a specific excision or inversion to facilitate efficient clonal isolation while minimizing hands-on cell culture time and effort (Fig. S9). In cases where the desired outcome is too rare for direct clonal isolation to be practical, ddXR provides an alternative option for sequential enrichment by the classical genetic technique of sib selection. Our group previously validated the use of ddPCR for isolation of iPSC clones with single nucleotide substitutions via sib selection^26^. The various modifications of ddXR thus provide additional tools to generate allelic series of clonal cell lines for functional analysis. Due to the inherent limitations of the method as described above, downstream validation methods should be performed to characterize clonal events in additional detail.

This method also provides a robust tool for evaluating the determining factors that promote excision and inversion. Based on the varying frequencies we observe across a wide range of linear DNA length, it is likely that many factors affect excision and inversion formation. In contrast to a previous study^3^, we did not observe a strong decrease in excision frequency with increasing length and observed no correlation between inversion frequency and length. The previous study reported nearly undetectable frequency of excisions larger than approximately 20 kb in length, as measured by clone genotyping in an immortalized murine cell line, while we were able to detect both excisions and inversions up to 172 kb in length at surprisingly high frequencies. Differences in the species and type of cell lines could partially explain the differing outcomes of our experiments. It is also probable that some very large deletions compromise cell viability and/or proliferation, which would prevent the cells from establishing clonal populations. Conversely, our method requires only a small amount of DNA and can be performed at any time point after editing, without expansion of the edited populations. It should therefore be possible to study editing outcomes even in non-proliferative cells, such as primary cells and various differentiated cells derived from iPSCs.

Rigorous studies of the repair of single DNA cleavages induced by CRISPR-Cas have begun to reveal predictable patterns that can be used to promote a specific repair outcome^35,36^. Until now, the lack of suitable methods has slowed the discovery of similar rules for excision-linked DNA repair. Our data contradict the hypothesis that linear distance is the primary factor driving the frequency of excision or inversion and ddXR enables systematic interrogation of other potential determinative variables including different nuclease enzymes, indel activity, PAM orientation, chromatin state and 3D DNA structure. The additional ability to measure precise repair and allele-specificity is particularly relevant to disease modeling, where targeted deletion of specific sequences allows for the detailed study of structure–function relationships, and for therapeutic editing. An exciting application of allele-specific excision is therapeutic inactivation of dominant disease alleles. In this regard, we focused our studies on three candidate loci containing genes that cause severe dominant inherited neuropathy (*HSPB1, MFN2 and NEFL).* The methods described here represent an important advance toward development of effective therapeutic editing strategies for these and other severe diseases.

## Methods

### iPSC culture and CRISPR editing

All iPSCs were cultured in StemFit Basic02 (Ajinomoto) on plates coated with matrigel (Corning) at 37 °C, 5% CO_2_ and 85% humidity. gRNAs were designed using CRISPOR^37^ and ordered as crRNA plus tracrRNA (Integrated DNA Technologies). Ribonucleoproteins (RNP) were prepared by complexing 104 pmol of each crRNA/tracrRNA duplex with 52 pmol of spCas9 protein (MacroLab) prior to transfection. Cells were transfected with RNPs using the Lonza 4D-Nucleofector X unit with pulse code DS138. After nucleofection, cells were cultured in media with Rock Inhibitor Y-27632 (Selleck Chemicals). Edited samples for ddXR assay were harvested 2–4 days post nucleofection without passaging. Clonal cell lines underwent two rounds of manual clone picking followed by expansion until an adequate number of cells for both cryopreservation and DNA extraction was obtained. DNA was extracted from non-clonal samples using the DNeasy Kit (Qiagen). DNA for clone screens was extracted by QuickExtract (Lucigen) or ethanol precipitation.

The majority of experiments were performed using an extensively characterized iPSC line from a healthy individual^38^ (WTC) that is the parental line for the Allen Institute Cell Collection (https://www.allencell.org/). Additional iPSC cell lines were used for specific experiments as follows: The inversion clone in Fig. 2 was derived from an *HSPB1* mutant line^18^. The excision clone in Fig. S2b and the precise excision clone in Fig. 4 were derived from an *MFN2* mutant line^30^. All experiments in Figs. 5 and S8 were performed in two unrelated *NEFL* mutant lines, patient 1 has been previously published^30^ and patient 2 is from an unrelated donor that was reprogrammed from peripheral blood mononuclear cells using the same episomal method as the *HSPB1* mutant line. Figure 5 includes data from an unrelated healthy control line^39^ (WTB) and from the *HSPB1* and *NEFL* patient 1 mutant lines. All lines had a normal karyotype, retained pluripotent morphology, and were capable of differentiation into multiple lineage types. Derivation and use of human iPSCs was approved and performed in accordance with the rules and regulations of the UCSF Committee on Human Research, San Francisco, CA (study #10-02521). All subjects provided informed consent prior to participation.

### ddPCR assays

ddPCR primer/ probe pairs were designed with the Primer Express software, using the MGB Quantification option. All custom probes were MGB-modified (Thermo Fisher Scientific) except for the *HPRT1* probes which were LNA-modified (Integrated DNA Technologies). FAM-conjugated probes were designed to match sequence on the outside of one of the excision gRNAs, approximately 20 bp away (Fig. 1a). One primer was placed just outside the probe from the cut site, ideally 40 bp away from the cut site. The second primer was placed just outside the second cut site. To detect inversions, the second primer was designed within the cut region pointing outwards adjacent to the second cut site. Ideally, amplicons should be between 100 and 150 bp. For repetitive or GC rich regions, we designed primer sets that produced amplicons up to 200 bp. Probes to detect precise excisions were designed to overlap the predicted junction, with the breakpoint in the center of the probe (Fig. 4a). The SNP probes used for allele-specific ddXR were designed using the Primer Express MGB Allele Discrimination option. A pair of VIC/FAM probes were designed for a SNP near, but not within, the excision. The SNP should be chosen as close as possible to the excision target site to minimize disruption to linkage caused by DNA shearing (Fig. S8). All reactions, except for plasmid-based precision assay validation and allele-specific ddXR, include a HEX-conjugated probe and primers to the RPP30 gene (Bio-Rad Laboratories) on chromosome 10, which encodes ribonuclease P/MRP subunit p30 and serves as an internal control to normalize the frequency of the gene editing outcome in question. A probe to any reference gene present in two copies can be used in place of RPP30. Sequences of gRNAs, PCR primers and probes are provided in Tables S2–S6.

All 25 uL ddPCR reactions were composed of 12.5 uL Supermix for Probes (no dUTP) (Bio-Rad Laboratories), 1.25 uL 20X reference assay, 50 ng DNA, 1.25 20X FAM assay for the target edit and water to 25 uL. Each 20X target probe mixture was made of 18 uM forward and reverse primer each and 5uM target probe. Droplets were generated using 20 uL reaction mixture and 70 uL oil with the QX200 Droplet Generator (Bio-Rad Laboratories). Droplets were transferred to a 96-well PCR plate, sealed and run on a C1000 Thermal Cycler with a deep-well block (Bio-Rad Laboratories). For samples with the 91 bp deletion, a restriction digest was performed prior to PCR amplification. 500 ng of DNA was treated with FastDigest BstXI in FastDigest Buffer (Thermo Fisher Scientific). Restriction digests were incubated at 37 °C for 60 min followed by heat inactivation at 65 °C for 20 min.

All ddPCR reactions were run under the following thermal cycling conditions: (1) 95 °C for 10 min; (2) 94 °C for 30 s; (3) 58 °C for 1 min; (4) steps 2 and 3 repeated 39 times; (5) 98 °C for 10 min.

All ddPCR runs were analyzed using the Bio-Rad QuantaSoft Pro Software. For inversion and excision rates, the value listed as “ratio” (excision or inversion to RPP30) in QuantaSoft was used. For allele-specific ddXR, the ratio of FAM+/FAM+ events and FAM+/VIC+ events were used to calculate the ratio of edited alleles.

### Clone genotyping

All clones were genotyped by PCR using primers placed outside the excision. PCR products were run on a 1% agarose gel to check for the presence of an excision band. For short excisions, the larger, unedited amplicon may be visible. For inversions, a primer 5′ to each gRNA site was used to detect inversions. The product is only amplified after the inversion occurs as it reverses to orientation of the second primer. All primer pairs were tested on unedited DNA control to confirm there was no PCR band prior to excision or inversion.

### Generation of genomic DNA standards

DNA from a pure, edited clone was mixed with unedited DNA of the same parent cell line. A serial dilution was performed, each with a total volume of 30 uL. The amount of edited DNA was decreased by a factor of four with each dilution with additional data points at 1.56, 0.39 and 0.09 (1:2 dilutions) for better resolution at lower concentrations. All dilutions were made and quantified in technical duplicates. For the 4.09 kb excision, the same DNA dilutions were used for standard curves for both the GOS and LOS assays. R^2^ and RMSE values were calculated using Prism.

### Fluorescent in-situ hybridization

FISH was performed by Cell Line Genetics Inc. (CLG) in Madison WI. A cryopreserved vial of unedited WTC cells and WTC cells edited with guides chr1:2 and chr1:4 were sent to CLG. CLG performed FISH on 200 cells for each sample using probe 1p36.22 (BAC clone RP11-1005H15 chr1:11821652–11999215) to detect the deleted region and probe 1p36.13 (BAC clone RP11-1062E1 chr1:17114168–17348163) as a reference. Cells from the same sample and passage were used to extract DNA for ddXR quantification.

## Supplementary information

Supplementary file
