# Supplementary material for: Rapid, precise quantification of large DNA excisions and inversions by ddPCR
Source: Sci Rep. 2020 Sep 10;10:14896. doi: 10.1038/s41598-020-71742-z (PMC7483445; doi:10.1038/s41598-020-71742-z)
Supplement: Supplementary file 1 — Supplementary file [file 41598_2020_71742_MOESM1_ESM.docx]

**Supplementary Data and Information for:**

**Rapid, precise quantification of large DNA excisions and inversions by ddPCR**

Hannah L. Watry^1,2^, Carissa M. Feliciano^1,3^, Ketrin Gjoni^1^, Gou Takahashi^4^,

Yuichiro Miyaoka^4^, Bruce R. Conklin^1,2,5,6^*, Luke M. Judge^1,3^*

^1^Gladstone Institute of Data Sciences and Biotechnology, San Francisco, CA., ^2^Innovative Genomics Institute, Berkeley, CA.

UCSF Departments of ^3^Pediatrics, ^5^Ophthalmology and ^6^Medicine, San Francisco, CA.

^4^Regenerative Medicine Project, Tokyo Metropolitan Institute of Medical Science, Tokyo, Japan

*corresponding authors

Luke M. Judge, luke.judge@gladstone.ucsf.edu

Bruce R. Conklin, bconklin@gladstone.ucsf.edu

**Supplementary Figures**

**Figure S1:** **Validation of ddXR for excision quantification at 2 additional loci using clonal iPSC lines.** A,B) Validation of clonal line with heterozygous 1.7 kb excision on chromosome 7 (A) and heterozygous 14 kb excision on chromosome 1 (B). PCR spanning excision produced expected bands in the mixed sample and clone. Control PCR spanning 5’ or 3’ cut site showed bands in the unedited, clone and mixed sample. C,D) ddXR quantification of 1.7 kb (C) and 14 kb (D) excision in negative controls (no DNA and unedited) and heterozygous clone and mixed sample. Values are mean of 3 replicates ± s.d.

**Figure S2: Evaluation of the directionality of the ddXR assay.** A) Diagram of 5’ and 3’ versions of the excision assay. B) Quantification of frequency of 14 kb excision in a clonal iPSC line using 5’ and 3’ assays. Values are averages of two replicates ± s.d. p-value = 0.2879. C) Diagram of 5’ and 3’ versions of the inversion assay. D) Quantification of frequency of inversion in polyclonal 14.7 and 4.1 kb edited samples using 5’ and 3’ assays. Values are averages of three replicates ± s.d. p-value = 0.4369 and 0.9152.

**Figure S3:** **Restriction digest eliminates false-positive signal when quantifying short excisions.** A) Schematic of restriction digest recommended for short excision samples. B) Representative 1D data plots from ddXR quantification of a 91 bp excision on chromosome 1 with and without restriction digest with BSTXI FD enzyme. C) Percentage of excisions detected in unedited and edited DNA with and without restriction digest, using the gating thresholds indicated by the pink bar in (B). Restriction digest effectively removed false positive signal caused by efficient amplification of non-excised alleles. This simple modification can be performed on any short excision using a restriction enzyme recognition sequence present within the excised region, but not elsewhere within the expected amplicon. Short inversions do not present the same challenge since the orientation of the primers does not allow amplification of unedited alleles, regardless of length.

**Figure S4:** **Excision quantification by loss of signal (LOS) ddPCR assay.**

A) Schematic of LOS ddPCR assay for excision quantification. B) Comparison of ddPCR quantification of standard samples with defined frequency of a 4.09 kb excision by LOS (red) and ddXR (blue) assays. Samples are the same as those shown in Fig 2e. RMSE for LOS = 4.161. RMSE for ddXR = 0.9924.

**Figure S5:** **Quantification of excision and inversion frequency across a range of linear chromosomal distances in multiple cell lines.** A) Stacked excision (blue) and inversion (red) rates of paired CRISPR-Cas9 ranging from 91 bp to 172 kb apart. Data are the same as presented in Fig. 3 and represent combination of experiments targeting three distinct loci each on distinct chromosomes. B,C) Dot plots of excisions (B) and inversions (C) measured in three different cell lines at three loci with varying linear distance between paired CRISPR-Cas9 target sites (14 unique combinations) ranging from 91bp to 34 kb apart. Color and shape of points indicate locus. Pearson correlation detected no correlation between frequency of excision and length, r = -0.1334, p = 0.6494 (A) or inversions and length, r = 0.362, p = 0.1311 (B). D) Stacked excision (blue) and inversion (red) rates (different method of representing the same data shown in B,C). E) Box plot comparing overall frequency of excisions and inversions across the dataset from B-D as percentage of total alleles. Whiskers extend to 10^th^ and 90^th^ percentile. Outliers are marked with black squares. Median_excision_ = 10.25%, Median_inversion_ = 5.045%, p = 0.0002.

**Figure S6:** **Validation of large mixed population excision rate by FISH.**

A) Comparison of quantification of 172 kb excision by ddXR and FISH. ddXR value is an mean of three replicate assays ± s.d. B,C) FISH result table and representative images from unedited (B) and edited (C) samples. Red Probe = Control. Green Probe= experimental (within excision). Cells with both alleles intact have 2 red and 2 green signals, cells with 1 green and 2 red represent a heterozygous excision event. C) Edited sample includes five cells with a single copy of both reference and experimental probes. While these could be FISH artifacts, it is possible that these cells had a larger than expected (>5 Mb) excision that ablated both the target sequence and the sequence recognized by the reference probe.

**Figure S7: Precise excision assay and characterization of precise and imprecise excision clones.** A) Sanger sequencing data from clonal iPSC lines with a 14kb excision on chromosome 1, one with a precise repair of the predicted excision junction (precise clone) and one with a 23bp insertion at the excision junction (imprecise clone). Probe designed to detect precise repair junction is highlighted in blue, with arrow indicating the junction between cut sites. BLAST alignment of the inserted sequence in the imprecise clone suggests that it could be derived from a foreign source of DNA. B-D) 2D plots of genomic DNA spiked with individual *HRPT1* excision reporter plasmids containing precise excision (B) and 1 bp deletion (C) or 1 bp mismatch (D).

**Figure S8:** **Validation of allele specific ddXR on unedited, clonal, polyclonal lines.** A-E) Additional representative 2D data plots from unedited control (A), clonal line with excision on WT allele (B), clonal line with excision on mutant allele (C), clonal line with homozygous excision (D) and edited population of cells homozygous for WT allele (E). Allele discrimination probes detect unedited alleles represented by single-positive FAM and VIC populations for WT allele (green) and mutant allele (blue). Excision on WT allele produces FAM-VIC double positive population (orange) while excision on mutant allele produces FAM-FAM double positive (purple). The small number of single-positive droplets in (B-E) are produced by shearing of DNA that disrupts the linkage between the SNP and excision site in a subset of templates. The source of this population is most apparent in (E), where the purple population is present, despite the cell line lacking the mutant allele. The ddXR excision probe produces a FAM signal that partially overlaps with the FAM-FAM double-positive signal. This single-positive FAM signal produced by shearing explains the relatively higher FAM-FAM measured signal in clones with excision on the wild-type allele. Decreasing distance between the SNP and the excision site is expected to minimize this shearing phenomenon. F) Quantification of allele-specific excision in edited population and clones, presented as proportion of FAM-VIC versus FAM-FAM signal. Excision occurred on both alleles, with 55.4% (Poisson range 61.2 – 49.6) linked to the mutant. Excision was identified on the mutant (mut) allele in three clones and on the wild-type (WT) allele in five, based on the predominant double-positive signal. * p < 0.0001 by two-way ANOVA and modified Tukey test.

**Figure S9: Schematic of clonal line derivation with or without ddXR.** Without ddXR clonal isolation is performed without knowing the rate of excision, necessitating the isolation and genotyping of additional clones. Thus, many weeks of labor are required to determine the optimal conditions. With ddXR, excision rates are quantified before clonal isolation, allowing only the optimal condition to be isolated and guiding the decision of number of clones to isolate. This greatly accelerates the choice of optimal conditions and saves many weeks of tissue culture.

**
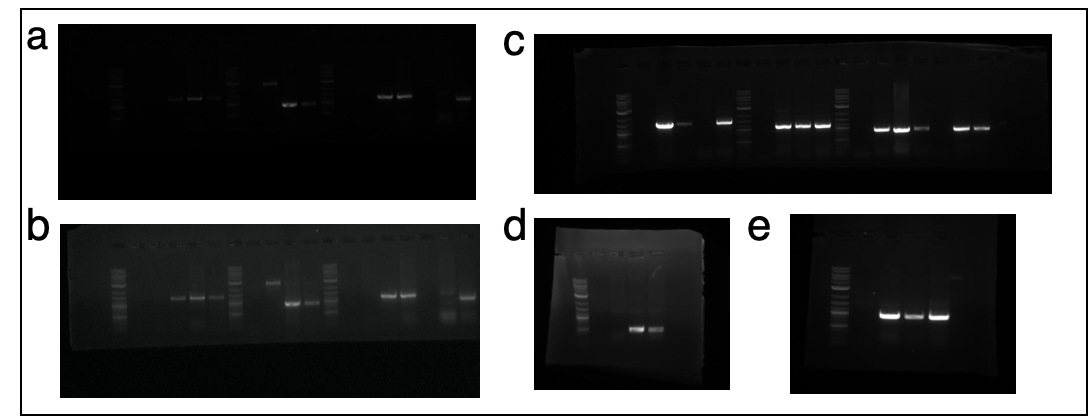
**

**Uncropped, unadjusted gel images for Fig. 2 and Fig S1.** A) Gel for excision junction PCR (upper) for 2a, S1a, S1b. B) Same as A, with brightness increased. C) Gel for cut site PCR (lower) for 2a, S1a, S1b. D) Gel for inversion junction PCR (Upper) for 2b. E) Gel for cut site PCR (lower) for 2b.

**Supplementary Tables**

|  | **Clones with excision/ inversion** | **Calculated % alleles edited** | **Measured % alleles edited by ddXR** |
| --- | --- | --- | --- |
| 1.7 kb Excision | 7/33 | 10.6% | 11.2% |
| 1.7 kb Inversion | 4/45 | 4.4% | 4.8% |
| 4.09 kb Excision | 3/30 | 5.0% | 3.5% |
| 14.7 kb Excision | 11/48 | 11.5% | 6.8% |

**Table S1:** Comparison of excision/ inversion rates determined by genotyping of clones and by ddXR.

| **gRNA ID** | **gRNA Sequence** |
| --- | --- |
| chr1:1 | TAGGACAACCGGCGAAAAAG |
| chr1:2 | GGAGGTATGACCCCCTACTT |
| chr1:3 | TAGTTCCGGTGGGCGTCGGC |
| chr1:4 | TAACCCAGTTTCCGCAGCGG |
| chr1:5 | TCAAGAAGTAGCTTTCGCCG |
| chr1:6 | ATAACCATCAGCCCGGATCT |
| chr1:7 | GTTTGAGCAGCACACGGTCC |
| chr1:8 | CCAGAAGCGGCACGCCTCGC |
| Chr1:9 | GATTCACCTGCTCGCTTAGG |
| chr8:1 | ACCCCTATTTATACGCCGGG |
| chr8:2* | TGGACCACGCTTATGAGTTC |
| chr8:3 | CAAGGCTTATCGAAATCATC |
| chr8:4 | TTTACGGTGATGGTGCGGCT |
| chr8:5 | TGCACGCAGCTCTTAGGGAT |
| chr8:6 | GCAGCTTTAATGCGGAACGC |
| chr8:7 | AGCTGGCGAAGCGGTCATTG |
| chr8:8 | GAGCTTTCTGCAAAGCCGCG |
| chr8:9 | GCATATTTCAGGCTAATGAG |
| chr8:10 | GGCGGCGTCCTTAGTAACGA |
| chr8:11 | AACTTGTTCGTTTGCGGGAT |
| chr8:11 | AGCGCGCTGCCCCCACTGGC |
| chr8:12 | AGCGCGCTGCCCCCACTGGA |
| chr8:13 | TTACGGTGATGGTGCGGCTA |
| chr8:14 | TTACGGTGATGGTGCGGCCA |
| chr7:1 | GCCTGCTAAAAATACCCGAC |
| chr7:2 | ATCTTTGCTCAGGCCCGTGC |
| chr7:3 | TGAATTCGAGAGCGCGACGC |
| chr7:4 | CGGGGGCGTGCGGTTGAAAC |
| chr7:5 | CGTTACATTACACACCGGGT |
| chr7:6** | CGACTCGAAGGTGACTGGGA |

**Table S2:** List of gRNAs used in excisions. gRNAs are identified by chromosome targeted.

*WTC and N98S patient cell line have one base pair mismatch (TGGTCCACGCTTATGAGTTC)

**Guide has one identical off target site on chromosome 9

| **gRNA Pairs** | **Length (bp)** |
| --- | --- |
| chr1:1/chr1:2 | 14,698 |
| chr1:1/chr1:3 | 91 |
| chr1:1/chr1:5 | 6,443 |
| chr1:1/chr1:6 | 14,732 |
| chr1:1/chr1:7 | 21,986 |
| chr1:4/chr1:2 | 172,082 |
| chr1:8/chr1:2 | 160,232 |
| chr1:9/chr1:2 | 171,946 |
| chr1:8/chr1:7 | 167,485 |
| chr8:1/chr8:2 | 4,087 |
| chr8:3/chr8:2 | 5,078 |
| chr8:4/chr8:2 | 4,511 |
| chr8:5/chr8:2 | 2,821 |
| chr8:6/chr8:2 | 2,689 |
| chr8:7/chr8:2 | 3,667 |
| chr8:9/chr8:2 | 46,433 |
| chr8:10/chr8:2 | 49,135 |
| chr8:1/chr8:8 | 14,017 |
| chr8:1/chr8:11 | 13,960 |
| chr8:11/ch8:14 | 374 |
| chr8:12/ch8:13 | 374 |
| chr7:1/chr7:2 | 1,750 |
| chr7:3/chr7:2 | 1934 |
| chr7:4/chr7:2 | 1254 |
| chr7:5/chr7:2 | 448 |
| chr7:6/chr7:2 | 292 |

**Table S3:** gRNA pairs and linear distance between target sites. 5’ guide is listed first.

|  | **F Primer** | **R Primer** |
| --- | --- | --- |
| chr1:1/chr1:2 Excision | GGCATTGATTCGCCGTTGTT | TGCTTTTCTCCCATCACGCT |
| chr8:1/chr8:2 Excision | TGAAAATGCCCTGCAAACCG | CCACCGAAGGTTCAAAGGAC |
| chr7:1/chr7:2 Excision | CCTCCTTAACAGAAGGACGGC | TGTCCTTGGGGTGTGCTGAAG |
| chr7:1/chr7:2 Inversion | CCTTAACAGAAGGACGGCCC | GCCACCTGTGTGTTCTTTTGAT |
| chr1:1/chr1:2 Cut Site | TGCTAGGCACACAGTGGTAG | TGCTTTTCTCCCATCACGCT |
| chr8:1/chr8:2 Cut Site | CAAAGTGGAAAGGACGACCG | CCAAGGAGCCAAGCCCTATC |
| chr7:1/chr7:2 Cut Site | CCTTAACAGAAGGACGGCCC | ACTCCCAATTCCTGAGCAAGG |

**Table S4:** Standard PCR primers used to genotype clones in Fig.2a,b, S1a,b and Table S1.

| **Event** | **Probe** | **F Primer (shared)** | **R1 Primer (excision)** | **R2 Primer (inversion)** |
| --- | --- | --- | --- | --- |
| chr1:1/chr1:2 (3’) | TGGGTGCAGGTAAGG | CAGGGTCAGGCTCTAGGACAA | AGCCATATCACTAGAGCAGAACTACAAT | CGGAGCCTCAAGCTGTCAAG |
| chr1:1/chr1:2 (5’) | AAGATTACAGAATGCAAATC | GCCTGGCCCTCTAGAGAACA | GACCTTACCTGCACCCAGTGA | TGGTCCTCTACTCCTCTGAACTATCA |
| chr1:1/chr1:3 | AAGATTACAGAATGCAAATC | GCCTGGCCCTCTAGAGAACA | TCGGACTCCCACTGCATCA | TTGACAGCTTGAGGCTCCG |
| chr1:1/chr1:5 | AAGATTACAGAATGCAAATC | GCCTGGCCCTCTAGAGAACA | GGAATTCCGACCCCAAAAGT | CCTTTGACTTCCTGGGATTGG |
| chr1:1/chr1:6 | AAGATTACAGAATGCAAATC | GCCTGGCCCTCTAGAGAACA | GCCACAAGATGGCGAGGATA | CCAGTTTCCTCACCTCCATCA |
| chr1:1/chr1:7 | AAGATTACAGAATGCAAATC | GCCTGGCCCTCTAGAGAACA | TGCAGGGAGTCCATGATGAG | TGTGTGTTCCAGGAGTGCATCT |
| chr1:4 /chr1:2 | TGGGTGCAGGTAAGG | GCCAGCATGATCCAAAGGA | AGCCATATCACTAGAGCAGAACTACAAT | AAACCCAGGAGTTCAAGGAGAGA |
| chr1:8/chr1:2 | TGGGTGCAGGTAAGG | CATTGGATTGGGCCACATCT | AGCCATATCACTAGAGCAGAACTACAAT | CTCACCATCAGTGTGACCATGA |
| chr1:9/chr1:2 | TGGGTGCAGGTAAGG | CTGGCAAGCCTCCTTGTTCT | AGCCATATCACTAGAGCAGAACTACAAT | GAGGCCACTTTCTGCAGGAAT |
| chr1:8/chr1:7 | ATTGCAGAGGCGGTTC | CATTGGATTGGGCCACATCT | TGCAGGGAGTCCATGATGAG | CTCACCATCAGTGTGACCATGA |
| chr8:1/chr8:2 (3’) | ACGGCAATGTGAATCA | GGCGTGCCGTGATCG | GTGAATTCATTTACTCATGTGGTGTTT | CCGTTCTGCCACCCCTATTT |
| chr8:1/chr8:2 (5’) | CAGGCTGCGTCAGG | GGCGTGCCGTGATCGA | N/A | TTGCTTGCAGAGTGGCTTTCT |
| chr8:3/chr8:2 | ACGGCAATGTGAATCA | CAGCGGCTTCCCTGAAAA | GTGAATTCATTTACTCATGTGGTGTTT | TCCGCTTTCCTCTTTTTACACAT |
| chr8:4/chr8:2 | ACGGCAATGTGAATCA | ATTAAAACTCTTCTCCATACATACTGCATAC | GTGAATTCATTTACTCATGTGGTGTTT | TCCCACCAGGAACCTCCTTA |
| chr8:5/chr8:2 | ACGGCAATGTGAATCA | GAGTTGGTGCGCCCAGAA | GTGAATTCATTTACTCATGTGGTGTTT | CCCACCCCTCCCACACA |
| chr8:6/chr8:2 | ACGGCAATGTGAATCA | GATAGGGCTTGGCTCCTTGG | GTGAATTCATTTACTCATGTGGTGTTT | GTGAATTCATTTACTCATGTGGTGTTT |
| chr8:7/chr8:2 | ACGGCAATGTGAATCA | ATCCGCACGCAGGAGAAG | GTGAATTCATTTACTCATGTGGTGTTT | TGCACGCGCTCGATGA |
| chr8:9/chr8:2 | ACGGCAATGTGAATCA | TCGTCATGACAAAGGTACTGTACACA | GTGAATTCATTTACTCATGTGGTGTTT | CTTGGGAAATGAGACAGCTGAAC |
| chr8:10/chr8:2 | ACGGCAATGTGAATCA | CGTATTCCGCCCAGAAAGG | GTGAATTCATTTACTCATGTGGTGTTT | GGGAGGTGGAGAAGCCAGTT |
| chr8:1/chr8:8 | GGCGTGCCGTGATCGA | CAGGCTGCGTCAGG | AAAGATCCAGCAAAACCCTGATT | TCGTTTGCGGGATGGG |
| chr8:1/chr8:11 | GGCGTGCCGTGATCGA | CAGGCTGCGTCAGG | GCGTGGCTCTACCAGCTCTAA | CATTTTGCACCTTAGGGGGA |
| Chr8:11/chr8:14  Chr8:12/chr8:13 | CAGGCTGCGTCAGG | GCCAGAAAGCTAGAAAGAAATTAAAACT | ACCCCTATTTATACGCCGGG | N/A |
| chr7:1/chr7:2 | ACACTGGTGTAGGTTGC | CAAACGGGTCATTGCCATTAA | TATGGATGTGAGTCAGCCTGTGT | GGCTCGGCTGCGCTTT |
| chr7:3/chr7:2 | ACACTGGTGTAGGTTGC | GCCTCTGCCACTTCTCAGTTG | TATGGATGTGAGTCAGCCTGTGT | GCCCTCATCTGGAACCTTCTC |
| chr7:4/chr7:2 | ACACTGGTGTAGGTTGC | TCCCTGGATGTCAACCACTTC | TATGGATGTGAGTCAGCCTGTGT | CCCGCACTCCCAATTCCT |
| chr7:5/chr7:2 | ACACTGGTGTAGGTTGC | GGACGAGCATGGCTACATCTC | TATGGATGTGAGTCAGCCTGTGT | CCTGGACGTGCAGAGAGGAA |
| chr7:6/chr7:2 | ACACTGGTGTAGGTTGC | AGCCACGCAGTCCAACGA | TATGGATGTGAGTCAGCCTGTGT | TCATCGGATTTTGCAGCTTCT |

**Table S5:** Probes and primers for all ddXR assays.

| **Assay** | **Probe** | **F Primer** | **R Primer** |
| --- | --- | --- | --- |
| LOS Assay | TACGAGCCGTACTACTC | CCGCCACCATGAGTTCCTT | CACGTAGCGCCGCTTGTAG |
| Chr1 Precision Assay | CGGCGAAACTTAG | GCCTGGCCCTCTAGAGAACA | GACCTTACCTGCACCCAGTGA |
| HPRT1 Excision Assay | CAGCCTCCAAAACTGTGAGA | ACCTTGCAGGTACCTTAATTTTG | GGAACAAAGCACCTCTGAGT |
| HPRT1 Precision Assay | ATAATAACCACATCATTTTATATGT |  |  |
| Allele Specific VIC (clones) | AGGACCTCAATGAC | CCAGGTAGCCGCCATCAG | GATGGCTCGGAGTGCTTCTG |
| Allele Specific FAM (clones) | CAGGACCTCAGTGAC |  |  |
| Allele Specific ViC | AGG ACC TCA ATG AC | CCTGGCCATGACCATCACTT | GGAGCCTAAGCGGGCATTA |
| Allele Specific FAM | CAG GAC CTC AGT GAC |  |  |

**Table S6:** Additional probes and primers used.

| **Plasmid** | **Cloned Sequence** |
| --- | --- |
| Precise | AGGCCTGAGATTGAAACCTACCTTGCAGGTACCTTAATTTTGGACTTCCCAGCCTCCAAAACTGTGAGAAATAAGTTTCTG  TTAAGTCACTCAGTCTGTGGTATTTTGTTATGGCAGCCTGAGCAGGTAGTTGTTCTTTCAGAAGGTGTTGATAATAACCAC  ATCATTTTATATGTATATATAAAAACGCATGCTGCCAAAGATAATTTATAAGAAAGACCATTGAATTTTTTTAAAAGTGAT  ATATATTCATTGAAAAAAATTTAGAATATATAGCAAAGCAATAAAGAACTAAATAAAATTGCTGTAACTCCTCTTTCAAAG |
| 1-bp Deletion | AGGCCTGAGATTGAAACCTACCTTGCAGGTACCTTAATTTTGGACTTCCCAGCCTCCAAAACTGTGAGAAATAAGTTTCTG  TTAAGTCACTCAGTCTGTGGTATTTTGTTATGGCAGCCTGAGCAGGTAGTTGTTCTTTCAGAAGGTGTTGATAATAACCAC  A-CATTTTATATGTATATATAAAAACGCATGCTGCCAAAGATAATTTATAAGAAAGACCATTGAATTTTTTTAAAAGTGAT  ATATATTCATTGAAAAAAATTTAGAATATATAGCAAAGCAATAAAGAACTAAATAAAATTGCTGTAACTCCTCTTTCAAAG |
| 1-bp Mismatch | AGGCCTGAGATTGAAACCTACCTTGCAGGTACCTTAATTTTGGACTTCCCAGCCTCCAAAACTGTGAGAAATAAGTTTCTG  TTAAGTCACTCAGTCTGTGGTATTTTGTTATGGCAGCCTGAGCAGGTAGTTGTTCTTTCAGAAGGTGTTGATAATAACCAC  A***A***CATTTTATATGTATATATAAAAACGCATGCTGCCAAAGATAATTTATAAGAAAGACCATTGAATTTTTTTAAAAGTGAT  ATATATTCATTGAAAAAAATTTAGAATATATAGCAAAGCAATAAAGAACTAAATAAAATTGCTGTAACTCCTCTTTCAAAG |

**Table S7:** *HRPT1* excision reporter plasmids used.

These 324-bp DNA fragments were cloned into pMD20 TA-cloning vector (Takara Bio) as the *HRPT1* excision reporter plasmids. The probe sequences are underlined. The deletion and mismatch are represented by a hyphen and a bold italic character, respectively.
